# Sequential Effects in Judgements of Attractiveness: The Influences of Face Race and Sex

**DOI:** 10.1371/journal.pone.0082226

**Published:** 2013-12-02

**Authors:** Robin S. S. Kramer, Alex L. Jones, Dinkar Sharma

**Affiliations:** 1 School of Psychology, Keynes College, University of Kent, Canterbury, Kent, United Kingdom; 2 School of Psychology, Adeilad Brigantia, Bangor University, Bangor, Gwynedd, United Kingdom; University of Nottingham Malaysia Campus, Malaysia

## Abstract

In perceptual decision-making, a person’s response on a given trial is influenced by their response on the immediately preceding trial. This sequential effect was initially demonstrated in psychophysical tasks, but has now been found in more complex, real-world judgements. The similarity of the current and previous stimuli determines the nature of the effect, with more similar items producing assimilation in judgements, while less similarity can cause a contrast effect. Previous research found assimilation in ratings of facial attractiveness, and here, we investigated whether this effect is influenced by the social categories of the faces presented. Over three experiments, participants rated the attractiveness of own- (White) and other-race (Chinese) faces of both sexes that appeared successively. Through blocking trials by race (Experiment 1), sex (Experiment 2), or both dimensions (Experiment 3), we could examine how sequential judgements were altered by the salience of different social categories in face sequences. For sequences that varied in sex alone, own-race faces showed significantly less opposite-sex assimilation (male and female faces perceived as dissimilar), while other-race faces showed equal assimilation for opposite- and same-sex sequences (male and female faces were not differentiated). For sequences that varied in race alone, categorisation by race resulted in no opposite-race assimilation for either sex of face (White and Chinese faces perceived as dissimilar). For sequences that varied in both race and sex, same-category assimilation was significantly greater than opposite-category. Our results suggest that the race of a face represents a superordinate category relative to sex. These findings demonstrate the importance of social categories when considering sequential judgements of faces, and also highlight a novel approach for investigating how multiple social dimensions interact during decision-making.

## Introduction

The order in which we are presented with items for judgement has a significant influence on those judgements. For example, the first and last items in a sequence show an advantage in later recall (*primacy* and *recency* effects, respectively [1]). Further, these sequence locations also prove advantageous during real-world decision-making like political voting, wine tasting, and competition jury evaluations [2-4]. In addition to the effects of this memory bias, judges also demonstrate a direct comparison effect, whereby the evaluation of the current item is influenced by the evaluation of the previous item. Using a large dataset of results from the ‘Idol’ television series, researchers found that singers who performed after a weak contestant were more likely to be evaluated poorly in comparison with those who performed after a strong contestant [5]. This assimilation effect was originally established within psychophysical tasks [6,7], but has more recently been found in such varied contexts as performance judgements of Olympic athletes [8], estimates of item prices [9], and ratings of essays [10].

In addition to assimilation, where the current judgement is drawn towards the previous judgement, research has also identified a contrast effect, where the opposite is true. When judges were told that gymnasts in a sequence shared the same nationality, their judgements showed assimilation, but when the nationalities were thought to differ, a contrast effect was found – higher scores were given after judges had previously seen weaker performances from athletes of a different nationality [8]. Evidence suggests that the degree of perceived similarity between two consecutive items determines whether assimilation (high perceived similarity) or contrast (low) takes place [11,12]. However, assimilation appears to be the default behaviour since people naturally search for similarities unless otherwise instructed [13].

In this paper, we investigate sequential effects in judgements of facial attractiveness. Only two research articles to date have considered this topic. As we might predict, the first demonstrated that judgements of attractiveness for a given face were indeed assimilated towards judgements of the preceding face [14]. However, while both male and female faces were rated in a randomly ordered sequence, only analyses incorporating all trials were presented. Importantly, trials might usefully be thought of as falling into one of two types: those where the preceding face and the current face are the same sex and those where they differ. While we may expect assimilation for the former, the lower perceived similarity in the latter type should reduce assimilation or even produce a contrast effect. In fact, this reduced assimilation was exactly what the authors found after carrying out additional analyses [15]. Here, we investigate how sequential effects may change depending on whether the previous and current faces fall into the same natural classification or not, and how these effects are modified when two overlapping classifications are present - sex and race.

### Own- and other-race faces

Within the face perception literature, it has been well-established that people are better at remembering own-race faces in comparison with faces of another race (for a meta-analysis, see 16). This other-race effect has generated several theories, although there are two main types of explanation. Perceptual expertise accounts suggest that perceivers have more experience with own-race faces, and this may lead to expert processing that is optimised to extract the relevant features for recognition from this specific set, resulting in improved accuracy and efficiency [17]. Alternatively, social categorisation accounts suggest that other-race faces may be categorised as those of out-group members, resulting in either a decrease in motivation to process other-race faces in terms of individual identity [18] or interference with coding individuating information because of the need to code race-specifying information [19,20]. Whether one or both of these explanations best accounts for the evidence to date, there is little doubt that our ability to recognise and differentiate other-race faces is comparatively worse [21].

Interestingly, an other-race advantage is found for race categorisation, with faster responses for other-race faces [22]. This may be explained by other-race faces forming a denser and more homogeneous cluster when encoded in a multidimensional space in comparison with own-race faces, due to suboptimal perceptual dimensions underlying their representation [23]. As such, although differentiation between other-race faces is more difficult, categorisation is made easier since activating one exemplar would partially activate a greater number of close neighbours and therefore the group membership.

Of particular relevance to the present paper is a finding by Rhodes, Locke, Ewing, and Evangelista [24]. Although unexpected and of secondary importance in their work, results showed that the other-race effect on recognition is increased when participants were required to rate the attractiveness of own- and other-race faces beforehand (relative to the no-rating control). This appeared to be driven by an improvement in recognition for own-race faces. From this, we might predict that the attractiveness ratings task used here will facilitate the deeper processing of own-race faces.

With regard to sequential effects, the fact that other-race faces are quickly categorised as such, and are perceived to be more similar to each other than own-race faces, suggests that a larger assimilation effect should be found with these faces. This would also be true if rating attractiveness produced deeper processing and more individuation of own-race faces [24]. In addition, given that we may automatically classify other-race faces as out-group faces, the perceived similarity between own- and other-race faces should be small. As such, we should expect a decrease in assimilation (or even a contrast effect) in trials where the current and previous faces differ in race.

### Categorising by race and sex

In reality, faces can be categorised by both race and sex. As such, the potential hierarchy of these two category labels may affect perceptions of similarity between individuals who differ on one or both. Previous evidence has suggested that for racial majorities, their day-to-day experiences tend to involve few interactions with other races but many with those of the opposite sex (of the same race). As such, sex should be a more accessible categorisation relative to race, and categorisation by sex does indeed produce faster responses [25]. Along similar lines, researchers have argued that faces are categorised based upon dimensions on which they deviate from a perceived norm – in America, for example, this perceived cultural norm may be “White male” [26]. The result is that faces are more likely to be categorised by race if they are not White, and by sex if they are female, and indeed there is some evidence in support of this hypothesis [27].

More recent evidence suggests that, in fact, race may be the superordinate category. Three-month-old White infants displayed a preference for female over male faces, but only when these faces are White rather than Asian [28]. In contrast, infant preferences for same- over other-race faces occurred for both male and female face pairings [29]. Taken together, these findings support the idea that faces are first categorised by race, and subsequent processing of sex is influenced by the result of this categorisation.

Support for this category hierarchy can also be found in the adult literature. Both White and Asian participants were more accurate at discriminating the sex of own- in comparison with other-race faces [30]. Using ERP techniques, researchers found that the N170, which is typically linked with the structural encoding of faces, was modulated by face race but not sex [31]. Further, although race and sex information are both activated early in processing, sensitivity to race appeared as early as 122ms in the N100, while sensitivity to sex emerged slightly later in the P200 [32].

Finally, when faces that fall into multiple categories are encountered, it may be that all applicable categories are activated in parallel and then compete (see 33). Indeed, recent research has provided support for this simultaneous activation [34]. Category salience may then play a role, along with chronic accessibility and temporary goal states, when determining the resultant categorisation [35]. Whether the goal of rating attractiveness facilitates the activation of race or sex remains unknown.

The lack of consensus in the literature makes predictions for sequential effects difficult. However, if we consider the ‘race precedes sex’ model as the best supported, we would predict that own- and other-race faces may differ in how much they are assimilated. If faces are categorised as sharing one’s own race, then one may subsequently process those faces using more individuating information, and this might lead to stronger effects of sub-categories like face sex. In terms of overall patterns, we might expect something akin to the three-month-old results above, where for each sex of face (i.e., faces are blocked by sex and vary in race), similar race effects are found. In contrast, for each race of face (i.e., faces are blocked by race and vary in sex), the effects of sex differ for own- versus other-race faces.

### The present paper

The aim of the present paper is to investigate the nature of sequential effects on attractiveness ratings. By considering separately those trials where the previous and current faces are the same sex/race and those where they differ, we can investigate the perceived similarity of faces within and between these categories with regard to attractiveness judgements. In Experiment 1, faces were blocked by race, which allowed us to explore the effect of sex for own- and other-race faces separately. In Experiment 2, faces were blocked by sex, allowing a comparison of race effects on male and female faces separately. Finally, in Experiment 3, faces differed in both race and sex within blocks. Here, we expected the strongest sequential effects, given that the previous and current faces had either two or no category dimensions in common.

## Experiments

### Experiment 1

In the first experiment, we investigated whether sequential effects differed for own- and other-race faces, with both males and females appearing within each sequence. We hypothesised that for both White and Chinese faces, assimilation would occur within-sex since shared category membership would be strong. However, given the apparent salience of race over sex as described above, combined with the differences in processing for other-race faces, we hypothesised that participants would assimilate between sex for other-race faces more than own-race faces. In other words, Chinese males and females would be seen as ‘Chinese’, while White males and females would be treated as ‘White males’ and ‘White females’.

### Method

#### Participants

Thirty-four students from the University of Kent (age range, 18-36 years; 19 females) participated in exchange for money or course credits. All participants self-reported as being White European.

#### Ethics statement

All of the studies reported in the current article were approved by the University of Kent Psychology Research Ethics Committee. All participants gave written informed consent prior to their participation.

#### Stimuli

Digital colour photographs of 100 White European (50 female) and 100 Chinese (50 female) students were selected for use in the current set of experiments from a larger database that had previously been collected at Bangor University. This subset were aged 18-30 years and provided consent for their images to be presented as stimuli. Actors were instructed to pose with a neutral expression and gaze directed toward the camera. Posture, lighting, and distance to the camera were held constant. Glasses were removed and hair was tied back where necessary. These photographs were then cropped below the chin, at the hairline, and at the left and right zygions. Images were approximately 400 x 500 pixels in size, and about 10 x 12.5cm on the screen.

### Procedure

Participants were shown all 200 images on the computer screen one at a time and instructed to rate the attractiveness of each face on a scale of 1 (very unattractive) to 10 (very attractive) using the mouse to make their responses. The experiment was self-paced, with no instruction to respond as quickly as possible, and viewing distance was not fixed.

White and Chinese faces were blocked separately, with sex varying within each block, and the order of block presentation was counterbalanced between participants. Trials appeared in a randomised order within each block for each participant. Between the two blocks, participants were allowed a short break.

### Results and discussion

First, we examined overall sequential effects by considering all trials for each race of face. For each block, participants’ ratings for the current face were correlated with their ratings of the face on the previous trial using Spearman’s rho. (The first trial in each block had no previous rating and so was excluded.) Fisher’s *r*-to-*z* transformation was then applied to these correlations in order to correct the skew in the distribution of *r*. Positive correlations would be an indication of an assimilation effect, whereas negative correlations indicate a contrast effect. We found assimilation (mean correlations significantly greater than zero) for both White, *M* = .17; *t*(33) = 6.49, *p* < .001, d = 1.11, and Chinese faces, *M* = .15; *t*(33) = 6.99, *p* < .001, d = 1.20. The magnitude of these results was similar to those of Kondo and colleagues [14].

In addition, for each block, we investigated whether trials prior to the one immediately preceding the current trial influenced attractiveness ratings using linear regression models that included three preceding trials. (Results for this and both subsequent experiments can be found in Table S1.) We found no significant influence of *n*-2 or *n*-3 trials with the exception of *n*-3 for Chinese faces, *M* = .03; *t*(33) = 2.24, *p* = .032, d = 0.38, which showed a small effect that was likely due to noise, given the lack of an *n*-2 effect. As such, replicating previous work [14], only the preceding trial produced a sizable assimilation effect.

In order to examine sequential effects while taking into account face categories, each participant’s trials within each block were separated into two trial types: the current face was preceded by a same-sex (‘male then male’, ‘female then female’) versus an opposite-sex (‘male then female’, ‘female then male’) face. For each of these two trial types, the participant’s ratings for the current face were then correlated with their ratings of the face on the previous trial and then transformed as above. (Again, the first trial in each block was excluded.) The descriptive statistics for this and both subsequent experiments can be found in Table S2.

**Figure 1:**
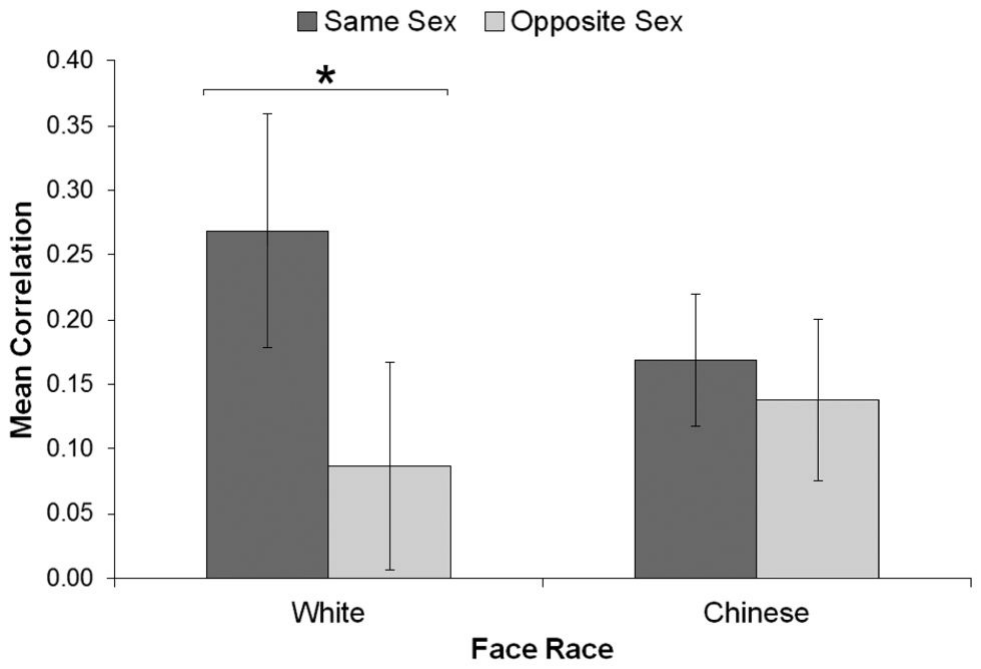
Mean correlation in Experiment 1 for each block (Face Race) and trial type. Error bars indicate 95% confidence interval and can be used to compare conditions to zero (i.e., error bars overlapping the zero line are not significantly different from zero). Same/opposite sex = same- or opposite-sex face on previous trial. * *p* < .01.

Correlations were entered into a three-way analysis of variance including Participant Sex (male, female), Face Race (White, Chinese), and Trial Type (same-sex, opposite-sex). Participant Sex varied between participants, while the other two factors varied within participants. As expected, there was a main effect of Trial Type, *F*(1, 32) = 6.42, *p* = .016, η_*p*_^2^ = .167. Trials where the preceding face was the same sex showed a higher assimilation (*M* = .22) than those where the preceding face was the opposite sex (*M* = .11). However, this was qualified by a significant Face Race x Trial Type interaction, *F*(1, 32) = 4.75, *p* = .037, η_*p*_^2^ = .129 (see Figure 1). For White faces, same-sex trials showed a significantly higher assimilation than opposite-sex trials, *F*(1, 32) = 7.46, *p* = .010, η_*p*_^2^ = .189, while these two trial types did not differ for Chinese faces, *F*(1, 32) = 0.72, *p* = .401, η_*p*_^2^ = .022. No other main effects or interactions were significant (all ps > .364).

Given that same-sex trials included both ‘male then male’ and ‘female then female’ trials, we also analysed these trial types separately. For White faces, both ‘male then male’, *M* = .23; *t*(33) = 3.87, *p* < .001, d = 0.66, and ‘female then female’ trials, *M* = .15; *t*(33) = 3.72, *p* < .001, d = 0.64, showed significant assimilation. Similarly, for Chinese faces, both ‘male then male’, *M* = .10; *t*(33) = 2.68, *p* = .011, d = 0.46, and ‘female then female’ trials, *M* = .12; *t*(33) = 3.49, *p* = .001, d = 0.60, showed significant assimilation. As such, our same-sex results were not limited to only one sex of face.

The interaction between Face Race and Trial Type demonstrated that the sex of other-race Chinese faces had no effect on sequential judgements of attractiveness (both trial types showed equivalent assimilation), while the sex of own-race White faces had a strong influence on the magnitude of the assimilation (same-sex trials assimilated significantly more than opposite-sex trials) [15]. This provides support for the hypothesis that faces were first categorised by race. Subsequently, how similar male and female faces were perceived to be depended upon this initial categorisation. For own-race faces only, perceived differences between males and females were substantial, almost eliminating assimilation in judgements for trials where male and female faces appeared straight after each other. This fits well with previous research demonstrating that viewers are more accurate in classifying own-race faces by sex in comparison with other-race faces [30], and that judging attractiveness can lead to the deeper (more individuating) processing of own-race faces [24].

By blocking the faces by race, one might predict that sex would become the most salient dimension for categorisation, and that this would be equally true for both blocks. However, in the Chinese block, participants appeared to classify all faces as ‘other race’ over and above identification of their sex. Interestingly, previous researchers found that when faces were blocked by race (same vs. other), a second, manufactured ingroup/outgroup categorisation (same-university vs. other-university) produced equivalent detriments in processing for outgroup members of both races [36]. These differences may be due to the nature of the tasks and questions considered (similarity and attractiveness in the current work versus configural processing of upright and inverted faces in the previous set of experiments), or the qualities of the dimensions featured (sex versus university affiliation). Further research might explore these empirically testable questions.

### Experiment 2

In the second experiment, we investigated whether sequential effects differed for female and male faces, with both White and Chinese faces appearing within each sequence. We hypothesised that for both female and male faces, assimilation would be larger for trials where the preceding face was of the same race (and therefore shared more similarities) in comparison with opposite-race trials. In line with previous research, we expected no differences in the effect of race for the female and male blocks.

### Method

#### Participants

A different group of 32 students from the University of Kent (age range, 18-32 years; 16 females) participated in exchange for money or course credits. All participants self-reported as being White European.

#### Stimuli

The same stimuli used in Experiment 1.

### Procedure

The procedure was identical to that used in Experiment 1 with one important difference: male and female faces were blocked separately, with race varying within each block. As before, the order of block presentation was counterbalanced between participants.

### Results and discussion

First, we examined overall sequential effects by considering all trials for each sex of face. We found assimilation (mean correlations significantly greater than zero) for both female, *M* = .18; *t*(31) = 7.97, *p* < .001, d = 1.41, and male faces, *M* = .18; *t*(31) = 7.53, *p* < .001, d = 1.33. The magnitude of these results was similar to those of Kondo and colleagues [14].

In addition, for each block, we investigated the influence of trials prior to the one immediately preceding the current trial. We found no significant influence of *n*-2 or *n*-3 trials with the exception of *n*-2 for male faces, *M* = .04; *t*(31) = 2.13, *p* = .041, d = 0.38, which showed a small effect that would require replication before we placed any confidence in it. As such, the preceding trial produced a sizable assimilation effect [14], while trials prior to this produced little or no effect.

**Figure 2:**
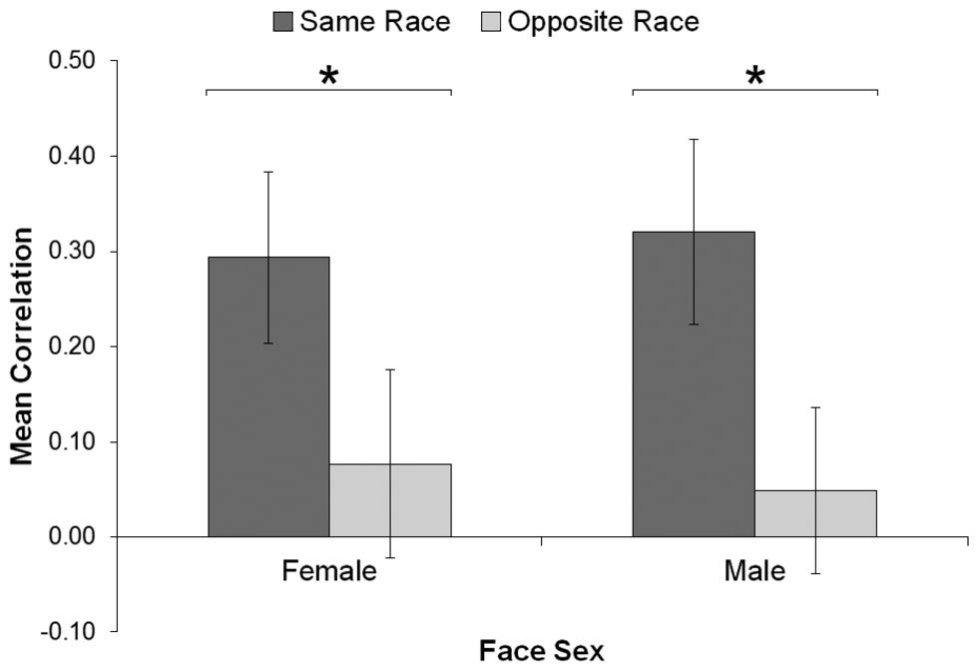
Mean correlation in Experiment 2 for each block (Face Sex) and trial type. Error bars indicate 95% confidence interval and can be used to compare conditions to zero (i.e., error bars overlapping the zero line are not significantly different from zero). Same/opposite race = same- or opposite-race face on previous trial. * *p* < .015.

Next, correlations taking into account face categories were calculated as in Experiment 1 except that trials were classified as those where the preceding and current faces were same-race (‘White then White’, ‘Chinese then Chinese’) versus opposite-race (‘White then Chinese’, ‘Chinese then White’). These correlations were entered into a three-way analysis of variance including Participant Sex (male, female), Face Sex (male, female), and Trial Type (same-race, opposite-race). Participant Sex varied between participants, while the other two factors varied within participants. As expected, there was a main effect of Trial Type, *F*(1, 30) = 11.43, *p* = .002, η_*p*_^2^ = .276 (see Figure 2). Trials where the preceding face was the same race showed a higher assimilation (*M* = .31) than those where the preceding face was the opposite race (*M* = .06). No other main effects or interactions were significant (all ps > .098).

Given that same-race trials included both ‘White then White’ and ‘Chinese then Chinese’ trials, we also analysed these trial types separately. For female faces, both ‘White then White’, *M* = .20; *t*(31) = 4.80, *p* < .001, d = 0.85, and ‘Chinese then Chinese’ trials, *M* = .16; *t*(31) = 3.64, *p* < .001, d = 0.64, showed significant assimilation. Similarly, for male faces, both ‘White then White’, *M* = .22; *t*(31) = 5.93, *p* < .001, d = 1.05, and ‘Chinese then Chinese’ trials, *M* = .22; *t*(31) = 4.07, *p* < .001, d = 0.72, showed significant assimilation. As such, our same-race results were not limited to only one race of face.

When participants rated White and Chinese faces of the same sex in a sequence, attractiveness judgements assimilated to the judgement on the previous trial more when the current and previous faces were the same race. In fact, when consecutive faces differed in race, participants showed no assimilation (mean correlations did not differ from zero in Figure 2). This held true for faces of both sexes equally (no main effect of Face Sex). Interestingly, we found no effects or interactions regarding Participant Sex. Although far less established than the other-race effect, a few studies have evidenced an ‘other-sex effect’ [37], whereby other-sex faces showed poorer recognition and discrimination. This would cause female participants, for example, to categorise male faces (of either race) as out-group members and subsequently perceive them as more similar to each other than in-group (female) faces. As such, we might see a pattern similar to Figure 1 where other-sex faces show no effect of Trial Type. However, we found no evidence of this effect in the current experiment, again supporting the idea that faces were initially classified by race, overshadowing any potential other-sex effects.

### Experiment 3

In the third experiment, we investigated sequential effects within a sequence of faces that differed on two dimensions at once. We hypothesised that assimilation would be significantly less on trials where both dimensions differed simultaneously in comparison with those where faces shared both dimensions. Indeed, by increasing the number of dimensions in which consecutive faces differed from one to two, we predicted that a contrast effect would emerge rather than merely a lack of assimilation [8].

### Method

#### Participants

A different group of 29 students from the University of Kent (age range, 18-34 years; 16 females) participated in exchange for money or course credits. All participants self-reported as being White European.

#### Stimuli

The same stimuli used in Experiment 1.

### Procedure

The procedure was identical to that used in Experiment 1 with one important difference: White female and Chinese male faces appeared in one block (WfCm), while White male and Chinese female faces appeared in the other (WmCf). As before, the order of block presentation was counterbalanced between participants.

### Results and discussion

First, we examined overall sequential effects by considering all trials for each block. We found assimilation (mean correlations significantly greater than zero) for both WfCm, *M* = .20; *t*(28) = 8.60, *p* < .001, d = 1.60, and WmCf, *M* = .21; *t*(28) = 5.32, *p* < .001, d = 0.99. The magnitude of these results was similar to those of Kondo and colleagues [14].

In addition, for each block, we investigated the influence of trials prior to the one immediately preceding the current trial. We found no significant influence of *n*-2 or *n*-3 trials with the exception of *n*-2 for WmCf sequences, *M* = .05; *t*(28) = 2.15, *p* = .040, d = 0.40, which showed a small effect that would require replication before we placed any confidence in it. As such, the preceding trial produced a sizable assimilation effect [14], while trials prior to this produced little or no effect.

**Figure 3:**
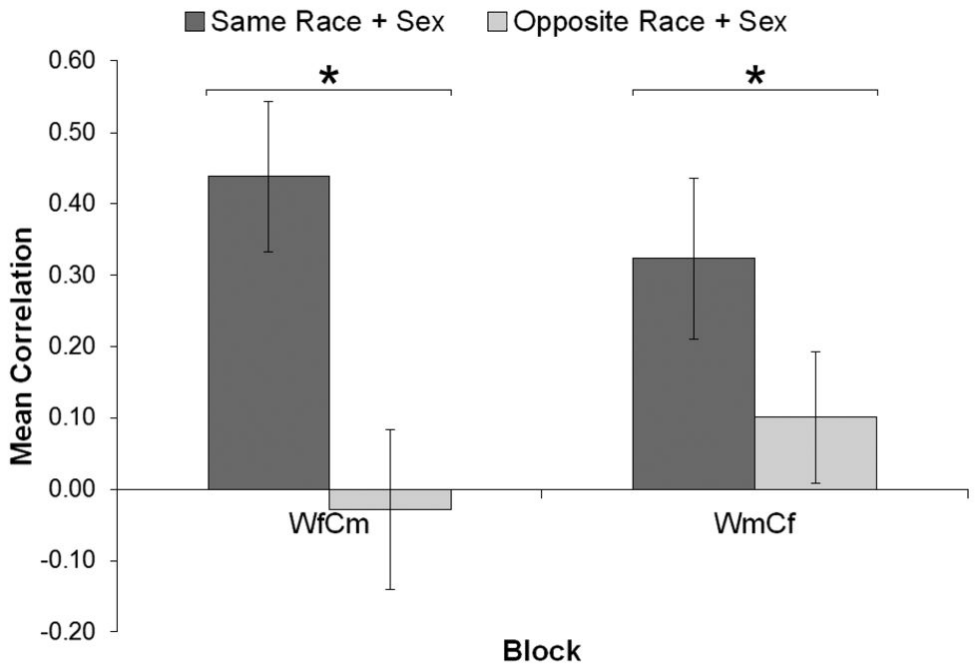
Mean correlation in Experiment 3 for each block and trial type. Error bars indicate 95% confidence interval and can be used to compare conditions to zero (i.e., error bars overlapping the zero line are not significantly different from zero). WfCm = White females and Chinese males, WmCf = White males and Chinese females. Same/opposite race + sex = face on previous trial is the same or opposite in both race and sex. * *p* < .001.

Next, correlations taking into account face categories were calculated as in Experiment 1 except that trials were classified as those where the preceding and current faces were the same race and sex (e.g., ‘White female then White female’) versus opposite race and sex (e.g., ‘White female then Chinese male’). These correlations were entered into a three-way analysis of variance including Participant Sex (male, female), Block (WfCm, WmCf), and Trial Type (same race and sex, opposite race and sex). Participant Sex varied between participants, while the other two factors varied within participants. As expected, there was a main effect of Trial Type, *F*(1, 27) = 33.55, *p* < .001, η_*p*_^2^ = .554. Trials where the preceding face was the same race and sex showed a higher assimilation (*M* = .38) than those where the preceding face was the opposite race and sex (*M* = .04). However, this was qualified by a significant Block x Trial Type interaction, *F*(1, 27) = 5.89, *p* = .022, η_*p*_^2^ = .179 (see Figure 3). This was caused by Trial Type having a larger effect in WfCm, *F*(1, 27) = 24.76, *p* < .001, η_*p*_^2^ = .478, in comparison with WmCf, *F*(1, 27) = 14.92, *p* = .001, η_*p*_^2^ = .356. No other main effects or interactions were significant (all ps > .506).

When participants rated faces of the same race and sex in a sequence, attractiveness judgements assimilated to the judgement on the previous trial more when the current and previous faces were the same race and sex. Further, this effect was stronger for sequences involving White females and Chinese males. Why participants perceived these two categories as more different (i.e., a larger decrease in the ‘opposite’ trials) than White males and Chinese females is unclear. Indeed, we might have expected the opposite result, given that Asians and females overlap in both facial phenotypes and stereotypes [38]. It may be that specifically considering attractiveness resulted in alterations to perceived similarity not found elsewhere, although further research is needed.

Although we found little or no assimilation when the preceding face differed in both sex and race, we failed to produce a contrast effect (e.g., higher ratings given after previously seeing lower attractiveness faces). The reason for this absence is unclear, and we discuss this issue further below.

## General Discussion

Over three experiments, we demonstrated the importance of social categories when considering sequential effects on judgements of facial attractiveness. Although people generally tend to assimilate ratings on any given trial towards those of the previous trial, our results highlighted the influence of category membership on such decisions. Further, the importance of race membership overshadowed that of sex classification, affecting subsequent judgements that necessarily involved similarity.

Our initial analyses for each experiment demonstrated that when all trials in a block were included (e.g., trials where two consecutive faces were the same sex and those where they were not), a significant assimilation occurred [14]. However, when the social categories of faces were taken into account, we found that the type of trial (consecutive faces were same-sex versus opposite-sex) influenced the nature of the resulting sequential effects. Indeed, this could represent the difference between a large assimilation of ratings and no measurable influence of the previous trial. As such, the importance of trial type in future research using sequential effects must not be overlooked.

Perceptions of attractiveness provide an interesting framework in which to consider sequential effects and social categories. Results from Experiment 1 suggest that faces were first categorised by race even though one might intuitively think that sex represented a more important category membership when considering attractiveness. When the average (heterosexual) person perceives attractiveness, we would predict that determination of opposite-sex membership is more important than opposite-race, given that faces of either race but not either sex might be seen as potential mates. Indeed, in Experiment 2, when participants rated sequences of faces of one sex (opposite-sex faces for one block), no assimilation was found when the previous and current face differed in race. That participants did not assimilate across race categories when judging attractiveness represents an interesting path for further research, and may be related to individual differences in racial prejudice.

By highlighting differences in category membership (e.g., nationality [8]), previous researchers have found a contrast effect (significantly negative correlation) in judgements. Although we found no contrast in the current set of experiments, our results demonstrated a decrease or elimination of assimilation. When race was salient (faces in a sequence only varied in race), participants did not assimilate across race categories. When sex membership was salient (faces only varied in sex), assimilation across sex categories was minimal for own-race faces [15]. When both race and sex were salient (Experiment 3), assimilation was either minimal or did not take place across faces that differed simultaneously in both categories. Why a contrast effect was not found, in particular in this final experiment where faces differed in two category memberships simultaneously, is unclear. It may be that asking participants to consider attractiveness increased the likelihood of assimilation due to the processes required to carry out such judgements. However, further work is required here.

Previous research has identified the perceived similarity of consecutive trials as a crucial factor in determining the influence of the previous trial on the current trial [11]. If a given face is more similar to the previous face, this may result in an increased perceptual fluency in processing of the current face, i.e., information regarding the current face is processed more easily immediately after processing a similar face. Easily processed stimuli are perceived as more likable [39] and so this account may also predict increased attractiveness for faces that appear after similar others. However, our pattern of results cannot be explained by this mechanism, given that positive correlations (assimilation in ratings) for similar faces are the result of both attractive and unattractive faces drawing subsequent judgements towards those already made. The fact that ratings can be either increased or decreased, depending on the nature of the previous face’s attractiveness, argues against a fluency account, which would predict only an increase following a similar face. As such, sequential effects in the current set of experiments are not the result of fluency of processing.

Can the current findings be explained in terms of differences in perceived attractiveness for own- versus other-race faces? We can address this issue by generating random ratings with specified means and ranges. If 100 Caucasian faces are rated randomly on a scale of 1-10, while 100 Chinese faces are rated randomly from 5-6, then the mean ratings are identical while the ranges differ. This might represent a lower sensitivity to other-race markers of attractiveness, or merely an increased perceived similarity between faces. We then calculate the correlation between current and previous trials for each race separately as in the experiments above. By carrying out this simulation for 10000 iterations (using MATLAB software), we find that the average assimilation effects for the Caucasian (*M* = -0.010) and Chinese faces (*M* = -0.010) do not differ from each other or from zero, which is to be expected since randomly generated ratings should not be influenced by previous trials. If we carry out this simulation again, but this time with Caucasian faces rated randomly from 6-10 and Chinese faces rated randomly from 1-5, then the mean ratings differ while the ranges remain identical. This is equivalent to perceiving other-race faces as generally less attractive (but still similarly varying). Again, we find that the average assimilation effects for the Caucasian (*M* = -0.012) and Chinese faces (*M* = -0.011) do not differ from each other or from zero. Overall, we conclude that general differences in own- versus other-race perceptions of attractiveness cannot account for our results. It is the relationship between ratings of the current and previous faces that produces assimilation effects rather than any potential differences in the scales that participants utilise.

Only judgements from White participants were collected in the current experiments. While this allowed us to specifically explore own- versus other-race influences on sequential effects, it would be important to consider other participant races in order to establish the generalisability of the current findings. For example, we would predict a race-reversed pattern of results with Chinese participants, where own-race (Chinese) faces show little or no assimilation between sexes while other-race (White) faces demonstrate assimilation within and between sexes. Indeed, initial support for this idea has been provided by results demonstrating that Japanese participants showed little assimilation between sexes for own-race faces [15]. In addition, we might find that experience with or exposure to other-race faces moderates these sequential effects, and the possibility for research in this area may prove fruitful.

The current set of experiments used a block design in order to highlight changes in a given sequence in either one (Experiments 1 and 2) or two (Experiment 3) category dimensions. However, in the real world, the faces that we are exposed to may differ in one or both dimensions within a single sequence. As such, it would be interesting to present faces of both races and both sexes within the same sequence, with the prediction that race membership would continue to overshadow sex membership.

By randomising the order of faces presented within a block, we were able to make sure that the particular face preceding any given image varied across participants. However, this also meant that the number of trials of each type was not equal. For example, in Experiment 1, analyses of our data show that participants were presented with an average of 1.5 more opposite-sex (compared with same-sex) trials in the block of White faces and 2.9 more opposite-sex trials in the Chinese block. Each block contained 100 trials and so we argue that these differences were negligible. However, future experiments might confirm that participants’ responses were not sensitive to small differences in the numbers of trials of each type by utilising predefined trial sequences that allow such numbers to be balanced.

While researchers in other areas have found an influence of trials prior to the one immediately preceding the current trial [6], research considering judgements of facial attractiveness has failed to find such an influence [14]. Here, we replicated this lack of a robust influence beyond the ‘*n*-1’ trial. However, due to the number of sequential trials used here, we were unable to consider the potential influence of specific sequence variants, e.g., is there an *n*-2 effect in ‘male-male-male’ sequences, and does this differ from ‘male-female-male’ sequences? With only approximately 12 trials per participant falling into each of these conditions, such analyses were not possible in the current set of experiments. Future research might tackle these specific questions using predefined sequences as discussed above.

One important issue to consider with regard to perceived similarity of own- versus other-race faces is its inherent dependence on actual similarity. While we know of no evidence that morphological variation in Chinese faces is less than that present in White faces, it is true that more general features like hair colour may show less variability. Although our photographs were cropped at the hairline, the Chinese faces all contained visible dark hair (with a few also showing light brown artificial highlights) while the White faces showed more varieties of hair colour. As such, one could argue that our Chinese photographs *were* actually more similar to each other. However, previous researchers have utilised cropped images where hair colour or style were not visible, and other-race effects remained significant [24,30]. As such, it is unlikely that this factor is able to account for our current findings.

The three experiments described here demonstrate the utility of sequential effects as a method for investigating social categorisation. By presenting faces (or other types of stimuli) using sequences and specifically considering the order of presentation, researchers are able to measure more implicitly the nature of perceived similarity. Here, we have focussed on the importance of race and sex, and whether these categories were perceived hierarchically when judging attractiveness. Other researchers might use this method for investigating facial categories like expression or age, for example. In addition, experimental manipulations of category salience, such as priming, could be employed in order to explore the potential malleability of social category hierarchies.

In conclusion, we demonstrate for the first time how sequential effects can be used in order to investigate the social categorisation of faces. Over three experiments, we found that when asked to judge the attractiveness of strangers, faces were first categorised by race and then by sex. Importantly, this order of categorisation has consequences, through differences in own- versus other-race face processing, for how much influence one face has on the next. Through altering the perceived similarity of those who differ in sex, we have shown that the other-race effect can play an important role in sequential judgements of attractiveness. Both the novelty and utility of this method of exploring social categorisation argue for its potential in future research into multiple category membership specifically and social perception more generally.

### Requests for Data

Data files are available upon request to the first author (RSSK).

## Supporting Information

Table S1 **Analysis of the influence of *n*-back trials.** For each participant, we predicted the current face’s rating (over all trials in a block) using the ratings given to the previous three faces in the sequence. Averaged partial regression coefficients (across all participants) for each face block were compared to zero. Sequences were not separated by trial type. * Significantly different at an uncorrected alpha level of .05; ** at .001.
(PDF)

Table S2 **A summary of the three experiments.** Mean correlations and standard deviations (SD) for all blocks and trial types, for each experiment.
(PDF)
