# Supplementary material for: Sequential Effects in Judgements of Attractiveness: The Influences of Face Race and Sex
Source: PLoS One. 2013 Dec 2;8(12):e82226. doi: 10.1371/journal.pone.0082226 (PMC3857852; doi:10.1371/journal.pone.0082226)
Supplement: Table S1 — Analysis of the influence of n-back trials. For each participant, we predicted the current face’s rating (over all trials in a block) using the ratings given to the previous three faces in the sequence. Averaged partial regression coefficients (across all participants) for each face block were compared to zero. Sequences were not separated by trial type. * Significantly different at an uncorrected alpha level of .05; ** at .001. (PDF) [file pone.0082226.s001.pdf]

Table S1. Analysis of the influence of *n*-back trials.

Experiment	Face Block	1-back	2-back	3-back
1	White	0.14**	-0.01	0.03
	Chinese	0.13**	0.01	0.03*
2	Female	0.15**	-0.01	0.02
	Male	0.13**	0.04*	0.03
3	White females + Chinese males	0.17**	0.01	0.00
	White males + Chinese females	0.14**	0.05*	0.02

For each participant, we predicted the current face's rating (over all trials in a block) using the ratings given to the previous three faces in the sequence. Averaged partial regression coefficients (across all participants) for each face block were compared to zero. Sequences were not separated by trial type. * Significantly different at an uncorrected alpha level of .05; ** at .001.
