# Supplementary material for: Sequential Effects in Judgements of Attractiveness: The Influences of Face Race and Sex
Source: PLoS One. 2013 Dec 2;8(12):e82226. doi: 10.1371/journal.pone.0082226 (PMC3857852; doi:10.1371/journal.pone.0082226)
Supplement: Table S2 — A summary of the three experiments. Mean correlations and standard deviations (SD) for all blocks and trial types, for each experiment. (PDF) [file pone.0082226.s002.pdf]

Table S2. A summary of the three experiments.

Experiment	Face Block	Trial Type	Mean	SD
1	White	Same sex	0.27	0.26
		Opposite sex	0.09	0.23
	Chinese	Same sex	0.17	0.15
		Opposite sex	0.14	0.18
2	Female	Same race	0.29	0.25
		Opposite race	0.08	0.27
	Male	Same race	0.32	0.27
		Opposite race	0.05	0.24
3	White females + Chinese males	Same race + sex	0.44	0.28
		Opposite race + sex	-0.03	0.29
	White males + Chinese females	Same race + sex	0.32	0.30
		Opposite race + sex	0.10	0.24

Mean correlations and standard deviations (SD) for all blocks and trial types, for each experiment.
